# Pen Pictures of Malaria

**Published:** 1899-11

**Authors:** Nelson W. Wilson

**Affiliations:** Acting Assistant Surgeon, U. S. Army


### PEN PICTURES OF MALARIA.

By NELSON W. WILSON, M. D.,
Acting Assistant Surgeon, U. S. Army.

IN PRESENTING these pen pictures, it is not the intention to in any way give a detailed clinical study of disease, its etiology, or treatment, but to present as briefly and as graphically as possible in limited space, a series of sketches of types of malaria, with here and there a case whose features were either interesting or puzzling, and in the most informal manner.

There are reasons why it was impossible to make a detailed and scientifically recorded study of the cases reported at the post hospital, Fort Porter, from the time the 13th U. S. Infantry arrived in Buffalo from Cuba, in September, 1898, to the time the command left for the Philippines, in April of this year. In the first place, there was not time to keep records or make detailed studies of individual cases, because of the great amount of work to be done and the lack of assistance and hospital accommodation. The hospital at Fort Porter contained eight beds when I reported there in September, 1898, and the day the regiment arrived in Buffalo, I had to use every cot I could get hold of to care for the seriously sick of the command. In addition to caring for those at the post many calls were received to visit soldiers of other commands than the 13th, who were confined in hospitals whose papers needed straightening out, such as extension of sick leaves, and the like; as well as soldiers ill in boarding houses who were entitled to medical attendance. This, in addition to the paper work required in the conduct of a post hospital, was sufficient to keep one on the jump from sick call in the early morning until late at night. The most one could do was to do his best under the circumstances.

That is why the cases referred to are presented purely as pen pictures of events in the course of an army post practice, and not given in properly tabulated, detailed form.

Dr Walter D. McCaw, captain and assistant surgeon, U.S. Army, assumed charge at the post hospital as post surgeon in October, and the work was then divided. Yet in spite of this we both had all we could do.

The first case came to me during the march of the 13th to Fort Porter from the depot. At North street a soldier fell out and tumbled in a heap on the sidewalk. He presented the perfect picture of a man overcome by heat. He was put into an ambulance and hurried to the Fort. On the way he recovered somewhat, and told me he was always seized the same way at the onset of a malarial attack. This collapse was followed about two hours later by the typical chill, fever and sweat, and the next morning he was apparently as well as could be expected of a man who had just campaigned in Cuba, and climbed San Juan hill in the front ranks, after lying in the trenches, half full of water and mud. He had a number of attacks after that, at irregular intervals, and each one began with utter and alarming collapse. He did not appear to mind the seizures very much either. They did not depress him as they did some of the men, and he bobbed up after passing through a paroxysm as serenely and as happy as though there had never been such a thing known as malaria. He was cured eventually by the treatment to be referred to later. There was not, *en passant*, a single failure that I can recall now, to stamp out malaria, when this treatment was followed.

In Cuba and at Montauk Point these men had been loaded with quinine in pill form. In Cuba they lived largely on a tinned beef diet, and in the majority of cases which reached Fort Porter there were stomach and intestinal disturbances of more or less serious type. These troubles were aggravated in many of the cases by the open-handed generosity and too kind hospitality of friends and relatives, who, in their endeavor to make up for the lack of food in Cuba, loaded up the returned soldiers with meat and drink of all varieties. There were quite as many reunion parties as there were returned soldiers, and these were invariably followed by a corresponding increase in the numerical strength of the sick report next morning. Right here, it will be of interest to note the effect of army discipline.

The old, seasoned soldier, while quite as ill with malaria as any of the recruits, did not suffer from the stomach difficulties, because he followed instructions and ate nothing but the food served at the post hospital.

One of the most interesting cases was that of a corporal, who had typical attacks of malaria. He would report at the hospital with a chill, and be put to bed. The usual fever followed and he seemed to come around to something like normal shape. The first time, it was 10 o'clock in the morning when, after a chill, he reported that he felt very comfortable and wanted to get out in the afternoon and go to duty. At 12.30 he was feeling about the same, but his entire body was a pale yellow. Later in the day his skin had assumed an orange color. He was not what one would call sick—he merely presented a series of color-changes which passed away in a comparatively short time. The entire condition was repeated during a subsequent attack.

There were two cases of severe epistaxis. Every time these men were brought into the hospital they had nose-bleed, which resisted all treatment save posterior plugging. In one of these cases the hemorrhage was so severe and persistent as to cause anxiety.

Two men with unmistakable malaria reported with eye-trouble. Their vision was dimmed. A careful cross-examination revealed that one man had for over thirty days been taking on an average two grams of quinine a day; the other could not give a clear idea of how much he had been taking, but it was considerable. He had taken it in pill form, in powder and solution. He had taken it in every form imaginable, and his was as clean-cut a case of cinchonism as one could wish for. They were ordered to stop taking quinine, and did so. They had severe malarial paxoxysms, but the toxic amblyopia was relieved and eventually their malaria was cured.

One of the officers at Fort Porter could tell hours before he had a chill or rise of temperature—in several instances he has foretold an attack forty-eight hours before it came on, and that too when he felt as well as he ever did in his life. He used to have an uncontrollable desire to stretch his right foot, and did so unconsciously. There was no yawning, nothing but the stretching of his foot; when that began, he prepared for a chill.

Taste of a peculiar and unmistakable character in two cases foretold attacks. One man had the taste of nuts in his mouth. This was invariably followed by a paroxysm. The other man expressed his taste thus: "I feel as if I had had an old copper cent in my mouth." In both cases chills and fever followed the unusual tastes.

An old-time soldier, one who had been in the service so long that he had become a philosopher, always reported at the hospital when he expected a chill. He foretold his attack by an itching on the abdomen. This was followed by a faint rash, and later the attack.

Hematuria occurred in three cases; not severe it is true, but always coincident with the attacks.

There were a great many cases where the malarial attack was preceded by severe gastric disturbances. These were most bothersome because it was hard to always differentiate between stomach disturbances caused by excesses of diet and drink and those caused by the malaria itself. There were some cases, however, where the statements of the soldiers could be replied upon implicitly, which were unquestionably caused by the malaria and preceded the attack. In other cases, the drinking of beer and whiskey were sufficient to produce an attack.

Two men presented startling pictures when they had their attacks. Their seizures were sudden, and profound. They were carried into the hospital in what, at first glance, seemed to be dying conditions. Respiration was slow and labored, pupils dilated, skin cold, eyes half closed and jaws relaxed, and they remained in this condition for some hours after their reception. It was their way of having malaria.

One of the most interesting phases of malaria which came under observation was that of a Rochester boy who had enlisted just as the regiment was starting for Cuba. He came back with a type of malaria which was wearisome, because it resisted all treatment. He was in bed and nothing which was done for him had any effect whatever. His temperature was up and remained up and he would not eat. I suggested to him one morning, as a sort of coaxer, that I wanted him to make an effort to eat, because as soon as he got strong enough to get out, I was going to give him a furlough for thirty days so he could go home. It was a chance shot at diagnosis, but it turned out to be a good one. His temperature came down, his appetite returned and in a week he went away to his mother, weak but happy. He told me after his return that he could not tell what he wanted when he was so sick, but that the minute I said "home" and promised him a furlough, he knew he was homesick. He had some malaria afterward, but it yielded to treatment.

As to treatment we stopped all quinine, after giving it in all sorts of ways, and Dr. McCaw suggested salicylate of cinchonidia in essence of pepsin, .33 to the teaspoonful of pepsin taken a few minutes before each meal. To this was added a Blaud pill, modified, after each meal, and aloin, strychnine and belladonna tablets at bedtime.

That this general plan of treatment for malaria was successful is shown by the fact that not a soldier at Fort Porter died, and that all who were under treatment for malaria have gone to the Philippines, with the exception of two or three, who were left behind because of expiration of enlistment, and one because of chronic gastritis.

Severe chills were broken by the administration of what the soldiers called "knock-out drops." This was made up as follows:

℞—Spts. chloroform.

Tr. opii . . . . . . . . . . . . . . . . . . . . . āā . . . 1.33

Spts. frument . . . . . . . . . . . . . . . . . . . 60.00

As soon as the efficacy of the cinchonidia mixture, which is now known among the soldiers as "the white medicine," was established, the Surgeon-General furnished it in unlimited quantities, and I understand it has been supplied for the Philippine service.

Fort Porter, N. Y.

